# Bioactive compounds from Chinese herbal plants for neurological health: mechanisms, pathways, and functional food applications

**DOI:** 10.3389/fnut.2025.1537363

**Published:** 2025-01-31

**Authors:** Wang Meng, Wang Chao, Zhao Kaiwei, Ma Sijia, Sang Jiajia, Xu Shijie

**Affiliations:** ^1^Institute of Basic Theory of Chinese Medicine, China Academy of Chinese Medical Sciences, Beijing, China; ^2^Jiangsu Provincial Hospital of Traditional Chinese Medicine, Nanjing, China

**Keywords:** bioactive compounds, Chinese herbal plants, traditional Chinese medicine, functional foods, neurological disorders

## Abstract

Neurological disorders pose significant global public health challenges, with a rising prevalence and complex pathophysiological mechanisms that impose substantial social and economic burdens. Traditional Chinese Medicine (TCM), with its holistic approach and multi-target effects, has gained increasing attention in the treatment of neurological diseases. This review explores bioactive compounds derived from Chinese herbal plants, focusing on their mechanisms of action, underlying pathways, and potential applications in functional food development. The review highlights the neuroprotective properties of flavonoids, alkaloids, polysaccharides, and polyphenols found in key TCM herbs such as *Scutellaria baicalensis*, *Salvia miltiorrhiza*, *Ligusticum chuanxiong*, and *Gastrodia elata*. These compounds have demonstrated significant anti-inflammatory, antioxidant, and neurogenic effects, making them promising candidates for the prevention and treatment of neurological conditions, including Alzheimer’s disease (AD), Parkinson’s disease (PD), and depression. Furthermore, the synergistic effects of TCM formulations targeting multiple signaling pathways offer advantages over single-target therapies, especially in combating neurodegenerative diseases. The review also discusses the challenges and future directions for integrating these bioactive compounds into functional foods and dietary supplements, aiming to improve neurological health and enhance clinical outcomes. Ultimately, this work aims to provide valuable insights into the potential of TCM-based interventions for promoting neurological well-being and addressing the global burden of neurological disorders.

## 1 Introduction

Neurological disorders represent a significant global public health issue, with their high prevalence and complex pathological mechanisms imposing a heavy burden on society and the economy (1–3). According to the Global Burden of Disease (GBD) study, it is estimated that in 2021, 3.4 billion people suffered from neurological disorders, accounting for 43.1% of the world’s population. These diseases led to approximately 11 million deaths (4). Common neurological disorders, such as Major Depressive Disorder (MDD) (5, 6), Alzheimer’s Disease (AD) (7–9), and Parkinson’s Disease (PD) (10, 11) not only severely affect the quality of life of patients but also place a significant strain on family and social resources. These conditions often come with long-term care requirements, leading to rising healthcare costs (12), and have a profound negative impact on patients’ mental health and social functioning (13, 14).

Despite certain advancements in modern medicine regarding the treatment of neurological disorders, current pharmacological therapies still face many challenges. On one hand, many drugs have significant side effects, including gastrointestinal discomfort (15), liver dysfunction (16) and even cognitive impairments (17), which often reduce patient adherence to treatment. On the other hand, some patients experience suboptimal responses to existing therapies, particularly in the middle and late stages of the diseases (18). Additionally, issues such as drug resistance are common, further limiting the long-term efficacy of medications (19). These problems underscore the urgent need for more effective, safe, and personalized treatment options.

In recent years, Traditional Chinese Medicine (TCM) has garnered increasing attention in the treatment of neurological disorders and is gradually gaining international recognition. Especially in the realm of neurological diseases (20), TCM has been recognized for its holistic regulatory effects (21), multitarget characteristics (22), and potential neuroprotective effects (23, 24). TCM intervenes in the pathological processes of neurological disorders by regulating the body’s overall balance, and is considered to have unique advantages in improving symptoms, delaying disease progression, and enhancing patients’ quality of life. For example, active ingredients in TCM, such as flavonoids, alkaloids, and polysaccharides, have been shown to exhibit significant effects in anti-inflammation (25), antioxidant activity (26), improving mitochondrial function (27), and regulating neurotransmitter levels (28). Furthermore, unlike single-target drugs, TCM often uses compound formulations to target multiple signaling pathways, achieving a comprehensive therapeutic effect.

This paper aims to systematically evaluate the potential applications of active ingredients derived from Chinese medicinal plants in the field of neurological diseases from the perspectives of their mechanisms of action, relevant experimental research evidence, and potential for development in dietary supplements and functional foods. By exploring the unique advantages and existing challenges of active ingredients in TCM for neurological health, this study aims to provide ideas for further research and clinical translation, as well as promote their development in the field of functional foods and nutritional interventions.

## 2 The basic evidence of TCM in the treatment of neurological diseases

### 2.1 Anti-inflammatory and antioxidant effects

Anti-inflammatory and antioxidant effects are key pathological mechanisms in the treatment of neurological diseases. Due to their multi-target regulatory properties, TCMs have shown significant advantages in this area. Experimental studies have found that TCM exhibits beneficial effects by intervening in key mechanisms of various neurological diseases. This section discusses the neuroprotective effects of representative Chinese medicines and their active compounds through modulation of inflammatory factors, antioxidant enzymes, and signaling pathways, based on experimental research, and systematically analyzes their potential applications in neurological diseases.

#### 2.1.1 Flavonoids: *Scutellaria baicalensis*

Research (29) has shown that active flavonoids extracted from *Scutellaria baicalensis*, namely Baicalin, Baicalein (BAI), and Wogonin, exert anti-inflammatory and antioxidant effects through multiple pathways. In terms of anti-inflammatory activity, Gong et al. (30) found that BAI reduced the generation of IL-1β and TNF-*α*, promoting the transition of microglial cells from the M1 to M2 phenotype, thus exhibiting anti-inflammatory effects. Wogonin reduces the activation of the STAT3 pathway, alleviates inflammation and neuronal apoptosis, and improves the function of spinal cord injury in rats (31). Jin et al. (32) demonstrated that administering103mg/kg of Baicalin for 33 days effectively suppressed the generation of pro-inflammatory cytokines such as IL-1β and IL-18 in APP/PS1 transgenic mice. This suppression of inflammatory cytokines also inhibited microglial cell activation, indicating the potential of BAI to mitigate neuroinflammation.

Regarding antioxidant effects, Yi et al. (33) suggested that BAI might alleviate oxidative stress by regulating key genes (such as AKT1, IL6, TP53), thus contributing to its antidepressant effects. Wogonin, on the other hand, increases the expression of phosphorylated Akt, Nrf2, and HO-1 through the PI3K/Akt/Nrf2/HO-1 pathway, while downregulating NOX2, caspase-3, Bax, and Bcl-2 proteins, thereby mitigating oxidative stress and neuronal apoptosis induced by traumatic brain injury (TBI) (34).

#### 2.1.2 Polyphenolic compounds: *Salvia miltiorrhiza* and *Rhodiola rosea*

The active compounds in *Salvia miltiorrhiza*—Salvianolic Acid A (SAA) and Salvianolic Acid B (SAB)—show synergistic effects in neuroprotection. Both SAA and SAB demonstrate significant antioxidant effects in multiple models. Experimental studies have shown that Salvianolic Acid (SA) activates the Nrf2/HO-1 pathway, scavenges reactive oxygen species (ROS), and improves mitochondrial function (35). In a chronic mild stress (CMS)-induced depression model, SAA combined with the antidepressant fluoxetine significantly improved depressive symptoms by reducing the expression of inflammatory cytokines TNF-*α* and IL-1β (36). Tan et al. (37) observed that Salvia and its key components SAA and SAB exhibited significant neuroprotective effects in an AD fruit fly model. In PC12 neuronal cell assays, SAA and SAB reduced cell death induced by Aβ42, diminished Aβ42 fibrillation, and improved activity in model fruit flies.

SA from *Rhodiola rosea* has been widely studied for its anti-inflammatory and antioxidant properties, particularly in scavenging free radicals and alleviating neuronal damage. In terms of anti-inflammatory effects, SA inhibits excessive activation of microglia and reduces the release of neuroinflammatory factors, particularly in an AD mouse model. SA reduces CD8+ T-cell infiltration and weakens microglial activation, mitigating the inflammatory response (38). Regarding antioxidant effects, SA significantly activates the Nrf2/GPX4 pathway, reducing ROS generation, inhibiting ferroptosis, and enhancing antioxidant enzyme activity to protect neurons from oxidative stress damage. Lu et al. (39) aimed to investigate the effects and mechanisms of SA on AD. Adult male C57BL/6 mice were administered 25 and 75 mg/kg of SA dissolved in physiological saline, once daily for 7 consecutive days. Compared to the control group, the model group exhibited significantly elevated levels of Tau, TNF-*α*, and IL-6, suggesting that neuronal damage and inflammation were aggravated in the PD mouse model. These results indicate that SA significantly alleviates neuronal damage and inflammatory responses in the mice.

#### 2.1.3 Polysaccharides: *Gastrodia elata*

The major components of *Gastrodia elata*, namely Gastrodia polysaccharide (NPGE) and Gastrodin, exhibit significant antioxidant and anti-inflammatory effects, reducing cerebral ischemia–reperfusion injury and alleviating PD symptoms. In terms of anti-inflammatory effects, studies have shown that administering NPGE for three weeks in MPTP-induced PD mice significantly inhibited the increase of glial fibrillary acidic protein (GFAP) in brain tissue and reduced levels of TNF-*α*, IL-1β, and IL-6 (40). Wang et al. (41) found that administering 100 mg/kg/d of Gastrodin for five consecutive days alleviated neuroinflammation in the hippocampus of mice, reducing the expression of TLR4/TRAF6/NF-κB pathway proteins, and suppressing the activation of microglia and astrocytes. Additionally, Gastrodin induced the shift of microglia from the pro-inflammatory M1 to the anti-inflammatory M2 phenotype, inhibited cell migration, and reduced phagocytosis.

Regarding antioxidant effects, NPGE has been shown to reduce ROS generation and downregulate IL-1β, IL-6, TNF-*α*, NLRP3, and HMGB1 levels in the MACO model of mice, thus suppressing neuroinflammation. This process may be mediated by the NRF2/HO-1 signaling pathway and NPGE’s anti-ferroptosis action (42).

#### 2.1.4 Alkaloid compounds: *Ligusticum chuanxiong*

The primary active component of *Ligusticum chuanxiong*, Tetramethylpyrazine (TMP), demonstrates significant anti-inflammatory and antioxidant properties in neuroprotection (43). TMP alleviates inflammation by inhibiting calcium ion overload, glutamate excitotoxicity, and oxidative stress. Liu et al. (44) reported that TMP, at a dose of 200 mg/kg/d, significantly reduced the expression of TNF receptor 1 (TNFR1), IκB-*α*, and NF-κB p65 proteins in spinal cord tissue after injury in rats. Enzyme-linked immunosorbent assay (ELISA) and immunohistochemistry (IHC) analysis indicated that TMP effectively inhibited the generation of TNF-α, IL-1β, and ROS, while increasing the expression of superoxide dismutase (SOD), improving axonal microstructure after spinal cord injury. Moreover, TMP reduced inflammation by inhibiting the activation of P2X7R and the NLRP3 inflammasome, lowering IL-1β and IL-18 levels and mitigating the inflammatory response following spinal cord injury (45). Danduga et al. (46) demonstrated that administering 60 mg/kg TMP significantly alleviated brain oxidative-nitrosative stress and neuroinflammation induced by pentylentetrazole, thus mitigating epileptic symptoms in rats. For AD, TMP regulates the SIRT1/Nrf2/HO-1 signaling pathway to reduce oxidative stress and improve diabetes-related cognitive impairments (47).

#### 2.1.5 Glycosides: *Rehmannia*

*Rehmannia* contains a variety of compounds, and its extracts exhibit significant anti-inflammatory and antioxidant effects, which can alleviate inflammation in neurological disorders such as multiple sclerosis (MS) (48). The most abundant component in *Rehmannia* is the iridoid glycosides, which, upon hydrolysis, may be converted into more active metabolites, making them one of the primary active constituents of *Rehmannia*. *Rehmannioside A* has advantages in neurodegeneration and antioxidant activity. It can induce the activation of Nrf2, ERK1/2, and CREB, while inhibiting the inflammatory responses mediated by JNK, MAPK, p38, and NF-κB (49). Fu et al. (50) established a cognitive impairment model in rats with middle cerebral artery occlusion (MCAO) for 14 days, followed by intraperitoneal injection of *Rehmannioside A* at a dose of 80 mg/kg. Compared to the model group, the *Rehmannioside A*-treated group showed significant improvements in cognitive dysfunction and neurological deficits, a reduction in the infarct area, and elevated levels of p-PI3K, p-Akt, nuclear Nrf2, HO-1, and SLC7A11 expression. These findings suggest that *Rehmannioside A* has neuroprotective effects, which may improve post-ischemic cognitive impairment through the inhibition of ferroptosis and activation of the PI3K/Akt/Nrf2 and SLC7A11/GPX4 signaling pathways.

#### 2.1.6 Supplementary Chinese medicines and active compounds

The primary chemical components of *Astragalus* membranaceus have been shown to possess antioxidant and hormone-like anti-inflammatory effects, particularly beneficial in the treatment of neurological diseases such as MS. Yong et al. (51) summarized that multiple active components of *Astragalus* regulate immune responses and suppress inflammation to exert therapeutic effects. *Curcuma longa* contains curcumin, which has attracted attention for its various pharmacological properties. Wei et al. (52) found that curcumin exhibited neuroprotective effects through its anti-inflammatory and antioxidant properties, proving useful in the treatment of various central nervous system diseases. Moreover, *Ziziphus jujuba*, a traditional Chinese medicinal fruit, also demonstrates antioxidant and anti-inflammatory properties with potential value in neuroprotection. Hua et al. (53) suggested that the fruit of *Ziziphus jujuba* has a variety of beneficial effects, including antioxidation and anti-inflammation, with therapeutic implications for central nervous system diseases.

This section reviews the progress in research on Chinese medicines and their active compounds with a focus on their anti-inflammatory and antioxidant effects. It highlights how flavonoids, polyphenolic compounds, polysaccharides, and alkaloids regulate inflammatory responses and oxidative stress to provide neuroprotective effects. Studies indicate that these components effectively reduce oxidative stress, modulate inflammatory cytokines, enhance antioxidant enzyme activity, and exert anti-inflammatory, antioxidant, and anti-apoptotic effects through various signaling pathways, such as Nrf2/HO-1, PI3K/Akt, and others (Appendix 1).

### 2.2 Neuron regeneration and neural plasticity

Neuron regeneration and neural plasticity are crucial for the recovery of neural function and the treatment of neurodegenerative diseases. Recent studies have shown that various active ingredients in TCM can significantly promote neuron regeneration and enhance neural network plasticity through multi-target and multi-pathway interactions. These compounds demonstrate notable effects in neural function repair by regulating neurotrophic factors, synaptic remodeling, and signal pathway activities. This section systematically reviews the research progress of active compounds from TCM, including polysaccharides, saponins, polyphenols, alkaloids, and triterpenoids, in neuron regeneration and neural plasticity.

#### 2.2.1 Polysaccharide compounds: *Lycium*, *Ganoderma lucidum*

*Lycium barbarum* polysaccharide (LBP) regulate the expression of proteins related to the IRS1/PI3K/AKT signaling pathway, reducing the accumulation of Aβ and the hyperphosphorylation of tau proteins in the brains of ICV-STZ mice, while upregulating the expression of synaptic-related proteins, thereby exerting neuroprotective effects. Additionally, LBP activates the ERK and PI3K/Akt signaling pathways to promote neuron growth and synaptic remodeling, and modulates neurotransmitter synthesis and metabolism to facilitate the functional recovery of neural networks (54). Xu et al. (55) demonstrated that LBP pretreatment alleviates oxidative stress and neurotransmitter imbalances induced by exposure to nonylphenol (NP) and octylphenol (OP). Furthermore, LBP significantly enhances neuroprotection via the p38-mediated SIRT1/MAOA and CREB/BDNF/TrkB pathways. *Ganoderma lucidum* polysaccharide (GLP) play significant roles in immune regulation and neural plasticity, reducing neuroinflammation and promoting neurotrophic factor secretion. For example, the antidepressant polysaccharide peptide (PGL) isolated from *Ganoderma lucidum* spores protects PC12 neurocytes from corticosterone toxicity and exerts antidepressant effects by upregulating BDNF expression and modulating key factors in the prefrontal cortex (56).

#### 2.2.2 Saponin compounds: *ginseng*, *Panax Notoginseng*, *Astragalus*

Since both *ginseng* and *Panax Notoginseng* contain Ginsenoside Rg1 (Rg1) (57), the following discussion will focus on the specific mechanism of Rg1. Research (57) has shown that Rg1 promotes the growth of retinal ganglion cell (RGC) axons and synaptic plasticity by activating the cAMP/PKA/CREB pathway. It also upregulates the expression of GAP43, Rac1, and PAX6, proteins closely related to neuron growth, while the PKA antagonist H89 can block this effect. Rg1 enhances glycolysis in RGCs, which may contribute to its neuroprotective effects. Rg1 can also directly transdifferentiate endogenous reactive astrocytes in rats into neurons and promote neuron regeneration after spinal cord injury, possibly by inhibiting the Notch/Stat3 signaling pathway (58). Liu et al. (59) found that continuous feeding of Rg1 (10 mg/kg and 20 mg/kg) for 14 days in demyelinated mice promotes functional recovery and enhances myelin regeneration. Rg1 increases the survival and proliferation of oligodendrocyte precursor cells (OPC) and induces the maturation of oligodendrocytes (OL), which is related to the differentiation signals transmitted by the GSK3*β*/β-Catenin pathway. Besides Rg1, *ginseng* also contains a significant amount of ginsenoside Rb1. A study (60) demonstrated that intraperitoneal injection of Rb1 (16.75 and 13.5 mg/kg) significantly improved memory impairment caused by chronic restraint stress (CRS). Rb1 also reduced the Bax:Bcl-2 ratio and the expression of cleaved caspase-3 and caspase-9, increased the levels of synaptophysin and postsynaptic density protein 95 (PSD95), and activated the BDNF/TrkB pathway in the hippocampus. Recent research on *Astragalus* primarily focuses on *Astragalus* saponin IV (AS-IV). Liu et al. (61) found that daily intraperitoneal injection of 40 mg/kg AS-IV for 4 weeks activated the BDNF–TrkB pathway, inhibited neuronal morphological damage and cognitive dysfunction in mice after radiation exposure, and exhibited neuroprotective effects.

#### 2.2.3 Polyphenol compounds: *Salvia miltiorrhiza*, *Rhodiola*, *Polygonum multiflorum*

Zhang et al. (62) found that oral administration of 10 mg/kg SAA or 5 mg/kg edaravone for 14 days significantly reduced infarct volume and neurological deficits in stroke rats, while improving pathological damage in the hippocampus and striatum. SAA promotes the proliferation, migration, and differentiation of neural stem/progenitor cells (NSPCs) and enhances axonal regeneration by activating the Wnt3a/GSK3*β*/β-catenin pathway, while inhibiting neuron apoptosis, showing superior neurogenesis effects compared to edaravone. Zheng et al. (63) showed that Salidroside (Sal) reduces neurological deficits and infarct volume in a middle cerebral artery occlusion/Ischemia–reperfusion (MCAO/IR) model, and protects against damage in an oxygen–glucose deprivation/reoxygenation (OGD/R) model. Sal promotes axonal growth by inducing autophagy, upregulating MAP2, GAP43, and PSD-95 protein expression; this effect was blocked by 3-MA, suggesting an autophagy-dependent mechanism. Additionally, Sal promotes motor function recovery in spinal cord injury mice by inhibiting the activation of JNK and STAT3 pathways, reducing the proliferation and polarization of A1 astrocytes, and promoting the differentiation and migration of neural stem cells (NSCs) to injured areas (64). Furthermore, resveratrol in *Polygonum multiflorum* plays a crucial role in synaptogenesis and neural repair. Amontree et al. (65) found that resveratrol activates SIRT1, reducing levels of MMP-9, MMP-2, and TIMP-1 in cerebrospinal fluid in AD patients. *In vitro* studies showed that resveratrol also inhibits the release of inflammation-related proteases from microglia and astrocytes.

#### 2.2.4 Alkaloid compounds: *Ligusticum chuanxiong*, pepper

The alkaloid compound mainly found in Chuanxiong is Tetramethylpyrazine (TMP). Research (66) showed that TMP pretreatment significantly alleviates learning and memory impairments induced by sevoflurane and improves neuronal damage, dendritic spine morphology, NMDAR2A, and PSD95 expression, as well as long-term potentiation (LTP) functionality. This suggests that Chuanxiong alkaloids may enhance hippocampal synaptic plasticity, mitigating neuronal damage and learning and memory impairments caused by sevoflurane. Hao et al. (67) found that treating rats with TMP (200 mg/kg/day) for 2 weeks significantly reduced the expression of miR-497-5p in spinal cord injury rats while upregulating EGFL7 levels. TMP inhibits apoptosis through this pathway, promoting angiogenesis, neural regeneration, and repair of neurological deficits. Nazifi et al. (68) found that piperine reduces the synaptic toxicity induced by STZ in the hippocampus, showing good neuroprotective potential.

#### 2.2.5 Triterpenoid compounds: *Ganoderma lucidum*

Research has shown (69) that Ganoderic Acid A (GAA) from *Ganoderma lucidum* regulates neuroimmune responses, increasing the expression of anti-inflammatory factor IL-4 and neurotrophic factor BDNF, while inhibiting the expression of pro-inflammatory factors IL-1β and IL-6, thus reducing microglial activation and astrocyte proliferation. The pharmacological or genetic deletion of farnesoid X receptor (FXR) would block the effects of GAA on myelin regeneration and motor function recovery in multiple sclerosis (MS) mice, indicating that GAA’s effects are FXR pathway-dependent. *Ganoderma lucidum* triterpenoids promote neurorepair and neural network functional recovery by increasing the expression of BDNF and NGF. Bupleurum saponins activate the BDNF/TrkB signaling pathway to improve stress-induced neuronal atrophy and synaptic plasticity disorders, thus alleviating depressive symptoms (70).

#### 2.2.6 Additional TCMs and active ingredients

Previous studies have shown that polysaccharide compounds, such as *Astragalus* polysaccharides, also play a significant role in enhancing neuron survival and neural plasticity (71). NPGE combined with electroacupuncture therapy can upregulate the expression of BDNF and SCF proteins in the tail shell nucleus of rats with focal cerebral ischemia. The combination of Polysaccharide of *Gastrodia Elata* Blume and electroacupuncture has a synergistic effect on the recovery from cerebral ischemia (72). Sun et al. (73) discovered that GLPs can inhibit hydrogen peroxide (H₂O₂)-induced cell apoptosis by reducing the expression of caspase-3, Bax, and Bim, while increasing the expression of Bcl-2. GLPs regulate the expression of apoptosis-related proteins and inhibit neuronal apoptosis induced by oxidative stress, demonstrating significant neuroprotective effects. However, no relevant studies have been published in recent years, so it has not been included in this review.

This section provides an overview of the research progress on TCMs and their active compounds in the areas of neuron regeneration and neural plasticity. It focuses on how compounds such as polysaccharides, saponins, polyphenols, alkaloids, and triterpenoids promote neuron regeneration and enhance neural network plasticity by regulating neurotrophic factors, synaptic remodeling, and key signaling pathways. Studies indicate that these compounds effectively improve neural function, increase neuron survival rates, and exert neuroprotective effects through multiple signaling pathways, including PI3K/Akt, cAMP/PKA/CREB, and Wnt/*β*-catenin (Appendix 2).

### 2.3 Mitochondrial function protection

#### 2.3.1 Polysaccharide compounds: *Lycium*

*Lycium* is rich in LBPs, which can reverse the light-induced suppression of the Nrf2/HO-1 signaling pathway in mice and cells. This compound helps mitigate cell apoptosis, oxidative stress, and mitochondrial damage, thereby offering neuroprotective effects (74).

#### 2.3.2 Polyphenolic compounds: *Salvia miltiorrhiza*, *Rhodiola*, *Polygonum multiflorum*, green tea

SAB from *Salvia miltiorrhiza* significantly alleviates mitochondrial damage caused by 1-methyl-4-phenylpyridinium (MPP+), inhibits oxidative damage, maintains mitochondrial membrane potential stability, reduces ROS production, enhances mitochondrial biogenesis, and increases the expression of NAD(P)H: quinone oxidoreductase. SAB restores mitochondrial function by activating AMPK and upregulating the expression of sirtuin 3, mitigating ROS-induced neuroinflammation, thus protecting neurons from MPP + -induced damage (75). Treatment with Sal also alleviates mitochondrial fission and fusion imbalance, autophagy, and reduces AMPK activity, promoting mitochondrial biogenesis in models of oxygen–glucose deprivation (OGD) or ischemia. Furthermore, it downregulates GRP75 expression, reducing mitochondrial calcium fluorescence intensity and MAM area induced by OGD (76). Another study found that SAB effectively protects mitochondrial morphology and function, likely by preventing excessive mitochondrial division (77). Resveratrol, through activation of the SIRT1-dependent PGC-1α/TFAM signaling pathway, promotes mitochondrial biogenesis, reduces mitochondrial-related inflammatory factors, and mitigates mitochondrial dysfunction and cell apoptosis induced by PrP 106–126 (78). At low concentrations, resveratrol activates the ERβ/NGB axis, promoting the accumulation of neuroglobin (NGB) in neurons, thus enhancing mitochondrial function and reducing H₂O₂-induced cell apoptosis, providing antioxidant protection (79). Zhao et al. (80) reported that intraperitoneal injection of 20 mg/kg resveratrol significantly increased cytochrome c oxidase (COX) activity, upregulated the mRNA levels of Cox5a, Cox6a1, and Cox7c, increased NAc ATP levels and mitochondrial quantity, and improved social deficits and anxiety-like behavior induced by adolescent social isolation. The epicatechin gallate (EGCG) in green tea regulates mitochondrial function, enhances synaptic plasticity, and reduces neuronal loss, improving memory deficits and behavioral performance. This may be related to the AMPK signaling pathway (81). Chen et al. (82) showed that intraperitoneal injection of 50 mg/kg EGCG for 1 month alleviated cognitive impairment, iron deposition, oxidative stress, and apoptosis induced by natural high-altitude hypoxia (HAH), promoting neuronal regeneration to combat chronic HAH-mediated neurodamage.

#### 2.3.3 Alkaloid compounds: *Ligusticum chuanxiong*, *Sophora*

Liu et al. (83) found that intraperitoneal injection of different concentrations of TMP (40, 80, 120, 160 mg/kg) once daily for 7 days significantly improved mitochondrial ultrastructure, elevated GPX4 levels, and reduced ACSL4 levels. Li et al. (84) discovered that ligustrazine (a compound from *Ligusticum chuanxiong*) enhances mitochondrial autophagy in a PPARγ-dependent manner, thereby increasing brain glucose metabolism and improving cognitive deficits in APP/PS1 transgenic mice. The main component of *Sophora flavescens*, matrine (MAT), significantly alleviates hippocampal ultrastructural damage caused by endoplasmic reticulum (ER) stress, including an increase in rough endoplasmic reticulum and mitochondria, and downregulates ER stress-related proteins (GRP78, CHOP, ATF6, Caspase-12), thereby improving spatial learning and memory deficits induced by diabetes.

#### 2.3.4 Saponin compounds: *ginseng*, *Astragalus*

Ni et al. (85) demonstrated that ginsenoside Rb1 protects mitochondrial function by inhibiting NADH dehydrogenase in mitochondrial complex I, reducing ROS generated by reverse electron transport. In conditioned astrocyte culture media, Rb1 not only protects mitochondrial function but also promotes mitochondrial transfer. When neurons are damaged under OGD/R conditions, Rb1 significantly improves mitochondrial membrane potential and oxygen consumption rate by enhancing astrocyte-mediated mitochondrial transfer, further supporting the hypothesis that Rb1 enhances neuronal tolerance via mitochondrial protection mechanisms. *Astragalus* saponins can alleviate Aβ pathology by reversing BDNF/TrkB signaling defects and mitochondrial dysfunction. They also reverse Aβ-induced cytotoxicity, apoptosis, mitochondrial stress, and synaptic toxicity, reducing the expression of p-TrkB, p-Akt, p-GSK3β, andβ-catenin in rat cortical neurons (86). Yin et al. (87) found that Astragaloside-IV inhibits the expression of Fas, FasL, Caspase-8, and Bax/Bcl-2 mRNA and downregulates apoptotic cytokines (Caspase-8, Bid, cleaved Caspase-3, Cyto C) following ischemia–reperfusion, suggesting that Astragaloside-IV might reduce cell apoptosis induced by ischemia–reperfusion through inhibition of key factors in both the death receptor and mitochondrial pathways.

#### 2.3.5 Flavonoid compounds: *Scutellaria baicalensis*, *Sophora japonica, Fructus Aurantii*

A study showed that the main component of *Scutellaria baicalensis*, Baicalin, improves memory by inhibiting PDE4, enhancing synaptic plasticity, preventing mitochondrial fragmentation, and rescuing dysfunction. Baicalin attenuates amyloid beta oligomers induced memory deficits and mitochondria fragmentation through regulation of PDE-PKA-drp1 signalling. Activates the PI3K/Akt/Nrf2 pathway, reducing ROS production in mitochondria, stabilizing mitochondrial membrane potential, and protecting brain cells from ischemic damage (88). *Sophora japonica* contains rich quercetin, which activates SIRT1 and the SIRT1/PGC-1α signaling pathway, promoting mitochondrial biogenesis and fusion, and reducing mitochondrial fission (89). Saberi-Hasanabadi et al. (90) showed that intraperitoneal injection of different doses of quercetin (50, 100, 200 mg/kg) in mice significantly decreased ROS, lipid peroxidation, and protein carbonylation in brain mitochondria, improving mitochondrial function and glutathione levels. The 200 mg/kg dose was more effective than the 50 and 100 mg/kg doses. Chandran et al. (91) found that pretreatment with the citrus flavonoid naringenin (100 mg/kg) in mice with cognitive impairment induced by methylmercury helped reduce oxidative load, thereby maintaining mitochondrial function and preventing neuronal cell death, ultimately improving cognitive deficits. Naringenin is one of the components in Fructus Aurantii.

#### 2.3.6 Other TCMs and active ingredients

Yang et al. (92) demonstrated that administration of 0.1 mg/g/day of Baji Tian oligosaccharides (MOO) *in vivo* and1.25, 2.5, and5mg/mL MOO *in vitro* increased the expression of Mfn2, thereby activating the PI3K/Akt/mTOR pathway to mediate mitochondrial autophagy, clearing damaged mitochondria in astrocytes. Jionoside A1 from *Rehmannia* is an iridoid glycoside that can reduce the consequences of ischemia/reperfusion injury by promoting mitochondrial autophagy mediated by Nix (NIP3-like protein X), thereby alleviating the symptoms of ischemic stroke (93).

This section reviews the research progress on the mitochondrial protective effects of active components in TCMs, focusing on how compounds such as polysaccharides, phenolics, alkaloids, saponins, and flavonoids improve mitochondrial function through various mechanisms. Studies indicate that these compounds effectively reduce oxidative stress, regulate mitochondrial autophagy, promote mitochondrial biogenesis, and protect neural cells through anti-inflammatory and anti-apoptotic actions (Appendix 3).

### 2.4 Integrative perspectives on TCM mechanisms for neuroprotection

In the treatment of neurological diseases, many TCMs and their active components exhibit significant neuroprotective effects through multi-target and multi-pathway interactions. Unlike traditional single-target drugs, the multiple mechanisms of TCM allow it to better address various pathological processes in complex neurological disorders, thus avoiding the common issue of drug resistance seen with single-target medications (94). In particular, the different roles and overlapping pathways of the same TCM component further enhance its potential in promoting neurological health, supporting the advantages of TCM formulas in the treatment of neurological diseases.

#### 2.4.1 Different actions of the same TCM and its effectiveness in treating neurological diseases

Taking *Ligusticum chuanxiong* as an example, its main active compound, TMP, has shown remarkable anti-inflammatory, antioxidant, and neuroplasticity-regulating effects. Studies have demonstrated that TMP significantly reduces neurodamage and improves neurological function by inhibiting oxidative stress, excitotoxicity due to glutamate, and calcium ion overload (66). Furthermore, TMP also plays a unique role in promoting neurogenesis and restoring neurological function, especially during the post-stroke recovery phase. This multi-target and multi-mechanism action highlights the significant potential of *Ligusticum chuanxiong* as a neuroprotective agent (67).

Similarly, *Scutellaria baicalensis* contains multiple neuroprotective compounds that act through diverse mechanisms. It exerts anti-inflammatory effects by reducing the production of inflammatory factors such as IL-1β and TNF-αand suppressing overactivation of microglia (30, 32). It also alleviates neurodamage by regulating oxidative stress-related genes such as AKT1, IL6, and TP53, demonstrating antidepressant and neuroprotective effects (33). These mechanisms contribute to its antidepressant and neuroprotective effects, positioning *Scutellaria baicalensis* as a promising therapeutic agent for neurodegenerative disorders like AD and PD.

#### 2.4.2 Pathway crosstalk and synergistic effects in neuroprotection

Active components of TCM exhibit unique advantages in treating neurological disorders through the crosstalk and overlap of multiple signaling pathways. For example, in *Salvia miltiorrhiza*, the active compounds SAA and SAB activate the Nrf2/HO-1 pathway to scavenge ROS, thereby improving mitochondrial function, significantly protecting neurons, and reducing oxidative stress (35). This action not only limits oxidative damage but also regulates mitochondrial function, enhancing neuronal tolerance to oxidative stress, and further alleviating neuroinflammation (36). Additionally, SAA and SAB promote neuronal proliferation, migration, and differentiation, enhancing neuroplasticity and supporting neurogenesis (37).

Moreover, *Rhodiola rosea* glycosides exhibit dual roles in anti-inflammation and antioxidation, reflecting synergistic pathway effects. Studies have shown that these glycosides activate the Nrf2/GPX4 pathway, reduce ROS generation, and inhibit ferroptosis molecules while enhancing antioxidant enzyme activity, thus protecting neurons from oxidative damage (64). Additionally, *Rhodiola* glycosides enhance neuronal network plasticity through autophagy mechanisms, promoting neuronal remodeling and recovery of neural functions. These actions not only play an important role in reducing neuronal death but also enhance the repair capacity of the nervous system by removing damaged organelles (63, 64).

Resveratrol also activates the SIRT1-dependent PGC-1α/TFAM signaling pathway to promote mitochondrial biogenesis, reduce mitochondria-associated inflammatory factors, and alleviate cell apoptosis and mitochondrial dysfunction caused by oxidative stress (78). Low concentrations of resveratrol activate the ERβ/NGB axis, enhancing neuroglobin (NGB) accumulation in neurons, further improving mitochondrial function and providing antioxidant protection (79).

Active compounds from different TCMs also show cross-talk within the same pathway. For example, the anti-inflammatory effect through the Nrf2 pathway is evident with Baicalin, which increases the phosphorylation of Akt, Nrf2, and HO-1, alleviating oxidative stress and neuronal apoptosis caused by traumatic brain injury (TBI) (34), as well as reducing mitochondrial ROS production and maintaining mitochondrial membrane potential stability (88). Similarly, Danshen phenolic acids scavenge ROS and improve mitochondrial function (35), while *Rehmannioside A* demonstrates neuroprotective and antioxidant properties by inhibiting the inflammatory responses mediated by JNK, MAPK, p38, and NF-κB (49), all through the same pathway. Additionally, Gastrodin reduces the expression of NF-κB pathway proteins, which alleviates neuroinflammation in the hippocampus of mice, decreasing the activation of microglial and astrocyte cells (41). Furthermore, *Ligusticum chuanxiong* extract, at a dose of 200 mg/kg/day, significantly reduces NF-κB protein expression in spinal cord tissue of rats with spinal cord injury, thus inhibiting inflammation and oxidative stress, and exerting neuroprotective effects (44).

These active components, through different signaling pathways and mechanisms, display synergistic effects in neuroprotection. In particular, in addressing the pathological processes of neurological diseases such as AD and PD, the modulation of multiple pathways provides a solid foundation for their efficacy. By activating various neuroprotective mechanisms, such as antioxidation, anti-inflammation, mitochondrial function protection, neuroplasticity enhancement, and promoting neuronal regeneration, these compounds can intervene in the pathological processes of neurological diseases in a multidimensional manner, thereby improving therapeutic outcomes and slowing disease progression. The crosstalk and overlap of these mechanisms highlight the advantages of single TCM active components in neuroprotection, especially when dealing with the multifaceted pathological mechanisms of neurological diseases, showing a broader and more profound therapeutic potential compared to single-target drugs.

#### 2.4.3 Advantages of TCM formulas: addressing drug resistance issues and clinical application prospects

Single-target drugs often face the issue of drug resistance in the treatment of neurological diseases, while TCM formulas, through multi-component and multi-target synergistic effects, can intervene in the different pathological processes of neurological diseases on multiple levels, thereby avoiding drug resistance and enhancing therapeutic efficacy. For example, the TCM components *Ligusticum chuanxiong*, *Scutellaria baicalensis*, and *Salvia miltiorrhiza* all intervene through anti-inflammatory, antioxidant, neurogenesis-promoting, and mitochondrial protection mechanisms, and can complement each other to enhance therapeutic effects in treating neurological diseases (31, 35, 66). This multi-target and multi-mechanism synergy ensures that TCM formulas can address pathological processes that a single drug cannot, while significantly improving the comprehensiveness and sustainability of the treatment (39).

Moreover, the application of TCM formulas allows for personalized treatment according to the specific conditions of individual patients. By adjusting the different components of TCM, the therapeutic effects can be finely tuned to meet the individualized needs of patients, ensuring optimal efficacy and safety. Compared to the single-target therapeutic strategies of Western medicine, the flexibility and multidimensional regulation provided by TCM formulas make them highly promising in the personalized treatment of neurological diseases (67, 75).

The multi-target and multi-mechanism synergistic effects of the same TCM components demonstrate significant neuroprotective effects, especially in the wide application of anti-inflammation, antioxidation, neuroplasticity regulation, and mitochondrial protection. The multidimensional effects of TCM formulas provide unique advantages in the treatment of neurological diseases. Future research should further explore their mechanisms, optimize treatment strategies, and promote their clinical application globally, offering more comprehensive and personalized treatment options for neurological health.

## 3 Dietary supplements and functional foods: current development and advantages of TCM plant sources

### 3.1 Global development status of dietary supplements and functional foods

With the increasing global aging population, neurodegenerative diseases such as AD and PD have garnered widespread attention (95). Dietary supplements and functional foods are increasingly recognized as complementary therapeutic options. From 2007 to 2018, the usage rate of dietary supplements (DS) among the U.S. population surveyed increased from 50 to 56%, while the usage of mineral nutrition (MN) products rose from 46 to 49% (96). Functional foods, which promote health and help prevent diseases associated with an aging society, have the potential to reduce the burden on public health infrastructure and offer self-treatment options (97). They also provide multiple health benefits, including antioxidant, anti-inflammatory, and immune-boosting effects, making them widely applied (98). In addition to their efficacy, regulatory reforms by the U.S. Food and Drug Administration (FDA) have stimulated the growth of global mobile health (mHealth) products, which has significantly contributed to the application of dietary supplements and functional foods (97, 99).

### 3.2 Current research and application of plant-based ingredients

Dietary supplements and functional foods widely incorporate plant-based ingredients, which typically possess antioxidant (100), anti-inflammatory (101), and neuroprotective (102) properties, particularly in improving neurological health. In addition to TCM ingredients, other plants also contain similar active compounds, such as resveratrol from grapes, which has antioxidant and anti-inflammatory effects that can alleviate neuroinflammation and combat neurodegenerative diseases (103). Anthocyanins from blueberries have significant antioxidant properties that scavenge free radicals, reduce neuroinflammation, and enhance cognitive function (100). Glucoraphanin in broccoli exhibits antioxidant and anti-inflammatory effects, improving neurological health. Quercetin in celery, through reducing free radical formation and inhibiting the release of inflammatory mediators, provides neuroprotective effects (104).

### 3.3 Advantages of TCM plant sources

#### 3.3.1 Multi-target effects of single TCM plants

Many single TCM plants exhibit multi-target therapeutic effects, especially in the field of neurological health, showing significant advantages. The complex chemical composition and multiple biological activities of TCM plant ingredients enable them to target multiple disease pathways, exerting synergistic effects. For example, the flavonoids in *Scutellaria baicalensis* have remarkable antioxidant and anti-inflammatory effects, improving cognitive function and slowing the progression of neurodegenerative diseases by inhibiting neuroinflammation and improving blood–brain barrier permeability (30, 88). Polysaccharides, carotenoids, and flavonoids in *Lycium barbarum* (goji berries) have antioxidant and anti-aging properties, protecting neurons and improving memory and cognitive function by modulating the immune system and enhancing neurotrophic factors (54, 55, 74). Active components like tanshinones in *Salvia miltiorrhiza* exhibit anti-inflammatory, antioxidant, and blood circulation-promoting effects, which help improve neural blood and oxygen supply, alleviating neurodegeneration, and show wide application potential in treating AD (62, 75, 105, 106). These multi-target effects of single TCM plants suggest that they not only act on multiple aspects of the nervous system but also collaborate through various mechanisms to enhance their overall therapeutic efficacy.

#### 3.3.2 TCM formulas: different target combinations and effects of various dosage forms

TCM formulations are an essential treatment modality in TCM, and through their combinations, they offer greater advantages in target selection and therapeutic efficacy. Research has shown that the main component of *Ligusticum chuanxiong*, tetramethylpyrazine, and *Astragalus* membranaceus, which contains astragaloside IV, can alter the polarization of astrocytes (A1/A2) through the Sirt1-NF-kappaB pathway, generating a synergistic effect on spinal cord injury (107). The main component of *Salvia miltiorrhiza*, SAIIA, when used in combination with *Ligusticum chuanxiong* pyrazine nanoemulsions, inhibits the MAPK/ERK/CREB signaling pathway, effectively alleviating cognitive impairment, oxidative stress, and neuronal apoptosis in AD rats, showing therapeutic effects on AD-induced cognitive dysfunction and neuronal damage (108).

Furthermore, TCM formulas can enhance the therapeutic effect by achieving synergistic enhancement or coordination among different components, while minimizing side effects. For example, Yangyin Tongnao granules (YYTN) significantly reduce TNF-*α* and Cyt-C levels and increase T-SOD in MCAO rats, exhibiting anti-inflammatory, anti-apoptotic, and antioxidant effects, with each of the individual ingredients showing effectiveness, though to varying degrees (109). The Kaixin San formula (KXS) improves cognitive and memory functions in AD rats via the Wnt/*β*-catenin signaling pathway (110), and also enhances mitochondrial autophagy and suppresses the NLRP3 inflammasome pathway, improving AD-related neuropathology and cognitive impairment in APP/PS mice (111). Additionally, it shows efficacy in preventing learning and memory impairment induced by 27-hydroxycholesterol (27-OHC) (112).

These examples demonstrate how the unique ability of TCM formulas to combine multiple active ingredients allows for the achievement of enhanced efficacy through synergistic action on various molecular targets, making them effective for addressing complex diseases such as neurodegeneration. The flexible combinations of TCM herbs and their dosage forms offer significant potential for improving treatment outcomes, reducing side effects, and providing more personalized therapeutic options for neurological disorders.

## 4 Opportunities and future development

### 4.1 Opportunities in current research

#### 4.1.1 Integration of modern science and TCM plant ingredients

With the advancement of molecular biology, genomics, and modern pharmacology, there is an increasing application of multi-omics technologies (such as transcriptomics, proteomics, and metabolomics) to study the mechanisms by which TCM plant ingredients affect the nervous system. For example, Wu et al. (113) utilized integrated proteomics and metabolomics to reveal that the Danggui Shaoyao San formula may exert therapeutic effects on AD by promoting the EM regulation of the GSK3β/PGC1α signaling pathway. Li et al. (114) employed serum metabolomics techniques to find that Polygala tenuifolia polysaccharides improved endogenous metabolites and gut microbiota disruption in SAMP8 mice caused by AD. Modern pharmacological research has found that the combination of certain TCMs with Western drugs can enhance therapeutic efficacy or mitigate side effects. For instance, Tabassum et al. (115) discovered that diazepam can enhance the anticonvulsant effects of peach extract, while the total alkaloids from *Pinellia ternata* can prevent seizures induced by arecoline in rat models (116). The application of these technologies not only deepens the understanding of the mechanisms underlying the effects of TCM but also provides important insights for clinical translation, especially in revealing the potential neuroprotective, regenerative, and reparative roles of plant-derived components. The application of these technologies not only deepens the understanding of the mechanisms underlying the effects of TCM but also provides important references for clinical translation, particularly in elucidating the potential neuroprotective, regenerative, and reparative roles of plant ingredients.

#### 4.1.2 Expanding market for dietary supplements

The global growth of the dietary supplement market provides new development opportunities for TCM plant ingredients (96, 97), particularly in the field of neuroprotection. As the global dietary supplement market expands rapidly, the potential of TCM plant ingredients as functional dietary supplements is continuously being explored. TCM plant ingredients have broad prospects for use in modern diets, particularly in meeting the multiple health demands of an aging society, such as antioxidant, anti-inflammatory, and immune-boosting effects. These natural plant-based bioactive compounds have significant biological activity that can effectively support brain health and delay the onset of neurodegenerative diseases. Medicinal food, a part of TCM, has accumulated thousands of years of practical experience, combining both medicinal properties and food ingredients to regulate the body and promote health (117). Medicinal food not only emphasizes the efficacy of the medicine but also takes into account the compatibility and taste of the ingredients, making the application of TCM more gentle and acceptable to patients both in terms of flavor and effectiveness. For example, pomegranates (118), a commonly used food-medicine dual-purpose plant, not only have remarkable anti-inflammatory and antioxidant effects but also enhance the nutritional value and functionality of daily meals. Moreover, modern scientific advancements have enabled better extraction, standardization, and application of some TCM active ingredients in contemporary diets (119, 120).

#### 4.1.3 Opportunities in precision nutrition and personalized interventions

In recent years, precision medicine and personalized nutrition have provided opportunities for individualized interventions with TCM plant ingredients. Precision medicine and personalized interventions involve tailoring health plans based on an individual’s genetic makeup, lifestyle, and health status (121–123). Combining TCM’s multi-target properties with personalized approaches can optimize the regulation of the nervous system according to individual needs, offering customized and precise treatments. This approach aligns closely with the principles of TCM, which emphasize individualized diagnosis and treatment.

#### 4.1.4 Opportunities in optimizing TCM dosage forms

Different dosage forms of TCM (such as decoctions, granules, capsules, etc.) vary in absorption rates, bioavailability, and pharmacological effects. By combining traditional formulations with modern technologies such as nanotechnology (124, 125) and controlled-release systems (125, 126), the bioavailability of TCM ingredients can be enhanced, improving their efficacy in promoting neural health. For example, several bio-materials are already used to mediate TCM ingredients, synergistically exerting antioxidant, anti-inflammatory, neuroprotective, and anti-apoptotic effects (127). Capsule forms can control drug release (128), while granules offer better solubility and absorption (129). Optimizing dosage forms will improve the clinical application efficiency and patient compliance with TCM therapies.

### 4.2 Future development directions and challenges

Although the research on the bioactive components of TCM plants is currently thriving, the mechanisms of many TCM plant compounds remain incompletely understood. Future studies are needed to elucidate their multi-target actions and intricate signaling pathways. Additionally, there is a significant lack of large-scale, long-term, and high-quality randomized controlled trials (RCTs). More clinical research is essential to validate the efficacy of TCM plant compounds as dietary supplements and functional foods. While some previous studies had raised concerns about the safety of herbal medicines (130), it is worth noting that the aforementioned TCMs are generally considered safe. Traditional TCM processing methods (Pao Zhi) have also enhanced the safety profile of these remedies. Furthermore, recent studies have provided a more comprehensive understanding of herbal medicines, with extensive animal and clinical experiments being conducted. These findings are gradually gaining recognition in the academic mainstream (131, 132). Nevertheless, as Yang et al. (130) have previously emphasized, before the widespread promotion of TCM-derived active ingredient formulations, it remains critical to rigorously evaluate their safety. To ensure the safety and efficacy of TCM plant compounds, it is imperative to establish more stringent standardization and quality control systems. These measures are essential to support the global adoption of TCM products. Research in these areas requires further strengthening in the future.

## 5 Summary

Plant-based bioactive compounds, especially those derived from TCM plants, exhibit unique advantages in areas such as anti-inflammatory, antioxidant, neurorepair, and mitochondrial protection, offering effective supplementary means for neurohealth interventions. TCM plant ingredients, as core components of dietary supplements and functional foods, have vast market potential and application prospects, meeting the growing concerns of modern society about neural health. As research deepens, particularly with the expansion of clinical studies, the application of TCM plant ingredients will provide new solutions for the prevention, treatment, and management of neurological diseases. The further promotion of standardization and global application will provide a more comprehensive and personalized treatment approach for neurohealth.
