# Supplementary material for: Bioactive compounds from Chinese herbal plants for neurological health: mechanisms, pathways, and functional food applications
Source: Front Nutr. 2025 Jan 31;12:1537363. doi: 10.3389/fnut.2025.1537363 (PMC11825344; doi:10.3389/fnut.2025.1537363)
Supplement: Supplementary file 1 [file Table_1.docx]

Appendice1:

| **Category** | **Chinese Medicine and Compounds** | **Mechanisms and Effects** | **References** |
| --- | --- | --- | --- |
| **Flavonoids** | *Scutellaria baicalensis*: Baicalin | Anti-inflammatory: Reduces IL-1β, TNF-α; modulates microglia. | (30, 32) |
|  | *Scutellaria baicalensis*: Wogonin | Antioxidant: Activates PI3K/Akt/Nrf2/HO-1; reduces ROS. | (33, 34) |
| **Polyphenolic Compounds** | *Salvia miltiorrhiza*: SAA, SAB | Antioxidant: Activates Nrf2/HO-1; reduces oxidative stress. | (35, 36) |
|  | *Rhodiola rosea*: Salidroside (SA) | Anti-inflammatory: Inhibits microglial activation. | (38) |
|  |  | Antioxidant: Activates Nrf2/GPX4; reduces ROS. | (39) |
| **Polysaccharides** | *Gastrodia elata*: NPGE | Anti-inflammatory: Inhibits TNF-α, IL-1β, GFAP expression. | (40, 41) |
|  | *Gastrodia elata*: Gastrodin | Antioxidant: Suppresses ROS; activates Nrf2/HO-1. | (42) |
| **Alkaloids** | *Ligusticum chuanxiong*: Tetramethylpyrazine (TMP) | Anti-inflammatory: Inhibits NLRP3 inflammasome activation. | (44, 45) |
|  |  | Antioxidant: Activates SIRT1/Nrf2/HO-1 signaling. | (46, 47) |
| **Glycosides** | *Rehmannia*: Rehmannioside A | Anti-inflammatory: Modulates MAPK, NF-κB pathways. | (49) |
|  |  | Antioxidant: Activates PI3K/Akt/Nrf2 and GPX4 pathways. | (50) |

Appendice2:

| **Category** | **Chinese Medicine and Compounds** | **Mechanisms and Effects** | **References** |
| --- | --- | --- | --- |
| **Polysaccharides** | *Lycium barbarum*: Polysaccharide (LBP) | Activates PI3K/Akt and ERK pathways; reduces Aβ and tau hyperphosphorylation; enhances synaptic remodeling. | (54, 55) |
|  | *Ganoderma lucidum*: Polysaccharide (GLP) | Upregulates BDNF; reduces corticosterone toxicity; modulates prefrontal cortex factors. | (56) |
| **Saponins** | *Ginseng*, *Panax Notoginseng*: Ginsenoside Rg1 | Activates cAMP/PKA/CREB pathway; promotes axonal growth and synaptic plasticity; enhances myelin regeneration. | (57, 58, 59) |
|  | *Ginseng*: Ginsenoside Rb1 | Activates BDNF/TrkB; reduces apoptosis-related proteins; improves memory. | (60) |
|  | *Astragalus*: Saponin IV | Activates BDNF/TrkB; protects against neuronal damage and cognitive dysfunction. | (61) |
| **Polyphenols** | *Salvia miltiorrhiza*: Salvianolic Acid A (SAA) | Activates Wnt3a/β-catenin pathway; enhances neural stem cell proliferation and axonal regeneration. | (62) |
|  | *Rhodiola*: Salidroside | Induces autophagy; promotes axonal growth and motor function recovery. | (63, 64) |
|  | *Polygonum multiflorum*: Resveratrol | Activates SIRT1; reduces inflammation-related proteases; promotes synaptogenesis. | (65) |
| **Alkaloids** | *Ligusticum chuanxiong*: Tetramethylpyrazine (TMP) | Enhances hippocampal synaptic plasticity; reduces apoptosis and neuronal damage. | (66, 67) |
|  | *Pepper*: Piperine | Protects hippocampal synaptic integrity; reduces neurotoxicity. | (68) |
| **Triterpenoids** | *Ganoderma lucidum*: Ganoderic Acid A (GAA) | Activates FXR pathway; increases BDNF/NGF expression; reduces neuroinflammation and astrocyte proliferation. | (69, 70) |
| **Additional TCMs** | *Astragalus polysaccharides* | Enhances BDNF/SCF expression; synergistic effect with electroacupuncture in ischemic recovery. | (71, 72) |
|  | *Gastrodia elata*: Polysaccharide (NPGE) | Reduces apoptosis-related proteins; demonstrates neuroprotective effects. | (73) |

Appendice3:

| **Category** | **Chinese Medicine and Compounds** | **Mechanisms and Effects** | **References** |
| --- | --- | --- | --- |
| **Polysaccharides** | *Lycium*: Lycium barbarum polysaccharides (LBP) | Activates Nrf2/HO-1 pathway; mitigates oxidative stress, apoptosis, and mitochondrial damage. | (74) |
| **Polyphenols** | *Salvia miltiorrhiza*: SAB | Activates AMPK and SIRT3; stabilizes mitochondrial membrane potential; reduces ROS and neuroinflammation. | (75,77) |
|  | *Rhodiola*: Salidroside (Sal) | Promotes mitochondrial biogenesis; reduces calcium overload and autophagy imbalance. | (76) |
|  | *Polygonum multiflorum*: Resveratrol | Activates SIRT1/PGC-1α; promotes mitochondrial biogenesis and reduces apoptosis and oxidative stress. | (78,79,80) |
|  | *Green Tea*: EGCG | Enhances mitochondrial function; alleviates cognitive deficits and oxidative stress. | (81, 82) |
| **Alkaloids** | *Ligusticum chuanxiong*: TMP | Improves mitochondrial ultrastructure; activates PPARγ-dependent autophagy; enhances glucose metabolism. | (83, 84) |
|  | *Sophora flavescens*: Matrine (MAT) | Reduces ER stress; alleviates mitochondrial damage and spatial memory deficits. | (85) |
| **Saponins** | *Ginseng*: Ginsenoside Rb1 | Protects mitochondrial function; enhances astrocyte-mediated mitochondrial transfer. | (85) |
|  | *Astragalus*: Astragaloside-IV | Reverses mitochondrial dysfunction; inhibits apoptotic pathways. | (86, 87) |
| **Flavonoids** | *Scutellaria baicalensis*: Baicalin | Regulates PDE4 and PI3K/Akt pathways; prevents mitochondrial fragmentation and dysfunction. | (88) |
|  | *Sophora japonica*: Quercetin | Activates SIRT1/PGC-1α; promotes mitochondrial fusion and reduces fission and oxidative stress. | (89, 90) |
|  | *Fructus Aurantii*: Naringenin | Maintains mitochondrial function; reduces oxidative load and cognitive deficits. | (91) |
| **Others** | *Baji Tian*: Oligosaccharides (MOO) | Activates PI3K/Akt/mTOR pathway; clears damaged mitochondria via mitochondrial autophagy. | (92) |
